# HIV-1 immune activation induces Siglec-1 expression and enhances viral *trans-*infection in blood and tissue myeloid cells

**DOI:** 10.1186/s12977-015-0160-x

**Published:** 2015-05-07

**Authors:** Maria Pino, Itziar Erkizia, Susana Benet, Elina Erikson, Maria Teresa Fernández-Figueras, Dolores Guerrero, Judith Dalmau, Dan Ouchi, Antonio Rausell, Angela Ciuffi, Oliver T Keppler, Amalio Telenti, Hans-Georg Kräusslich, Javier Martinez-Picado, Nuria Izquierdo-Useros

**Affiliations:** AIDS Research Institute IrsiCaixa, Institut d’Investigació en Ciències de la Salut Germans Trias i Pujol IGTP, Universitat Autònoma de Barcelona, Badalona, Spain; Institute of Medical Virology, National Reference Center for Retroviruses, University of Frankfurt, Frankfurt, Germany; Pathology Department, University Hospital Germans Trias i Pujol (HUGTIP), Badalona, Spain; Otorhinolaryngology Department, HUGTIP, Badalona, Spain; Institute of Microbiology, University Hospital Center and University of Lausanne, Lausanne, Switzerland; Swiss Institute of Bioinformatics (SIB) - Vital-IT, Lausanne, Switzerland; Current address: The J. Craig Venter Institute, La Jolla, CA USA; Department of Infectious Diseases, Virology, Universitätsklinikum Heidelberg, Heidelberg, Germany; Institució Catalana de Recerca i Estudis Avançats ICREA, Barcelona, Spain; University of Vic-Central University of Catalonia (UVic-UCC), Vic, Spain

**Keywords:** Dendritic cells, HIV-1, *Trans*-infection, Siglec-1

## Abstract

**Background:**

Myeloid cells are key players in the recognition and response of the host against invading viruses. Paradoxically, upon HIV-1 infection, myeloid cells might also promote viral pathogenesis through *trans*-infection, a mechanism that promotes HIV-1 transmission to target cells via viral capture and storage. The receptor Siglec-1 (CD169) potently enhances HIV-1 *trans*-infection and is regulated by immune activating signals present throughout the course of HIV-1 infection, such as interferon α (IFNα).

**Results:**

Here we show that IFNα-activated dendritic cells, monocytes and macrophages have an enhanced ability to capture and *trans*-infect HIV-1 via Siglec-1 recognition of viral membrane gangliosides. Monocytes from untreated HIV-1-infected individuals *trans*-infect HIV-1 via Siglec-1, but this capacity diminishes after effective antiretroviral treatment. Furthermore, Siglec-1 is expressed on myeloid cells residing in lymphoid tissues, where it can mediate viral *trans*-infection.

**Conclusions:**

Siglec-1 on myeloid cells could fuel novel CD4^+^ T-cell infections and contribute to HIV-1 dissemination *in vivo*.

**Electronic supplementary material:**

The online version of this article (doi:10.1186/s12977-015-0160-x) contains supplementary material, which is available to authorized users.

## Background

Antigen presenting cells of the myeloid lineage, such as dendritic cells (DCs), monocytes and macrophages, initiate antiviral immune responses and are crucial to control invading viruses. On the other hand, myeloid cells may also be productively infected with HIV-1 and thus promote pathogenesis. Compared to activated CD4^+^ T cells, the permissivity of myeloid cells for HIV-1 is limited [1-3]. This is largely due to restriction by the cellular factor SAMHD1 in these cells [4,5]. Nevertheless, myeloid cells can contribute to HIV-1 dissemination through the alternative pathway of *trans*-infection of CD4^+^ T cells [6,7]. This mechanism involves HIV-1 capture and uptake by myeloid cells and the subsequent release of trapped viruses at a cell-to-cell contact zone, the infectious synapse, that facilitates infection of CD4^+^ T cells [8].

We and others have previously shown that HIV-1 *trans*-infection requires the sialic acid binding Ig-like lectin 1 (Siglec-1, Sialoadhesin or CD169) [9,10]. Siglec-1 is expressed on the surface of DCs and other myeloid cells [11,12] and recognizes sialyllactose molecules exposed on HIV-1 membrane gangliosides [13,14]. Siglec-1 expression on myeloid cells is induced by activating signals that are present upon acute and chronic immune activation, which is observed in HIV-1-infected patients [15,16]. Various pro-inflammatory factors associated with HIV-1 disease progression stimulate Siglec-1 expression on myeloid cells. Lipopolysaccharide (LPS) is a component of the bacterial cell wall that is significantly increased in chronically HIV-1-infected individuals owing to the depletion of gastrointestinal CD4^+^ T cells, which causes systemic translocation of bacteria from the intestinal lumen [17]. *In vitro*, LPS is able to induce Siglec-1 expression on DCs, potently enhancing their capacity to *trans*-infect HIV-1 [9]. Another factor is interferon alpha (IFNα) that exerts antiviral effects on HIV-1 replication [10,18], but also serves as a marker of poor clinical prognosis during late-stage disease [19,20]. Although IFNα is produced in large quantities by plasmacytoid DCs after HIV-1 challenge [21,22] the key cells responsible for sustained IFNα production *in vivo* are currently under intensive reexamination [23,24]. Regardless of its source, IFNα potently induces the *in vitro* expression of Siglec-1 on myeloid cells [10,18].

Siglec-1 up-regulation by immune activating signals during HIV-1 infection could play an important role by allowing myeloid *trans*-infection of multiple target cells. This function could be particularly relevant in lymphoid tissues, the major sites of HIV-1 replication, where myeloid cells migrate and repeatedly establish interactions with CD4^+^ T cells [25-27], the primary targets of productive HIV-1 infection. In the study described here, we investigated whether Siglec-1 expression on myeloid cells can be induced during chronic HIV-1 infection and thus contribute to systemic viral dissemination. We found that Siglec-1 on myeloid cells is up-regulated *in vitro* by IFNα, allowing for HIV-1 capture and transmission. *In vivo*, HIV-1 infection enhanced Siglec-1 expression on peripheral blood monocytes, but diminished after effective antiretroviral treatment, reducing the *trans*-infection capacity of monocytes. Moreover, Siglec-1 is present on myeloid cells from lymphoid tissues, where it can mediate HIV-1 *trans*-infection.

## Results

### Siglec-1 mediates HIV-1 capture by IFNα-treated myeloid cells

**Figure 1:**
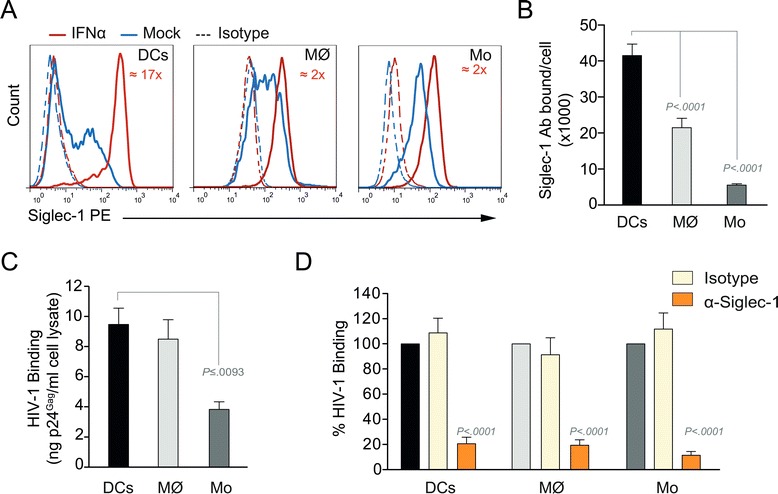
Siglec-1 mediates HIV-1 capture by IFNα-treated myeloid cells. **A**. Representative profiles of Siglec-1 staining in distinct myeloid cells cultured with or without 1000 U/ml of IFNα and assessed by FACS. Staining of matched-isotype control is also shown. The mean fold increase in fluorescence after IFNα treatment of cells derived from three donors is shown in red numbers. **B**. Mean number of Siglec-1 antibody binding sites per cell displayed by different myeloid cells exposed to 1000 U/ml of IFNα for 48 h and assessed by quantitative FACS analysis. Data show mean values and SEM from four experiments including cells from 12 donors. **C**. Comparative binding of HIV-1_NL4–3_ to different myeloid cells previously exposed to 1000 U/ml of IFNα for 48 h. Cells were cultured with HIV-1_NL4–3_ for 4 h at 4°C, washed and lysed to measure p24^Gag^ by ELISA. Data show mean values and SEM from two experiments including cells from six donors. **D**. Relative binding of HIV-1_NL4–3_ by different IFNα-treated myeloid cells that had been pre-incubated with 10 μg/ml of the indicated mAbs before HIV-1 exposure for 4 h at 4°C as described in **C**. To compare the effect of the mAbs in different myeloid cells, values were normalized to the level of HIV-1 binding by mock-treated cells (set to 100%). Data show mean values and SEM from two experiments including cells from six donors. Statistical differences were assessed with a paired t test in **B** and **C**, and with a one sample t-test in **D**.

Siglec-1 expression on the surface of myeloid cells can be stimulated by IFNα [10,18] an antiviral cytokine released by pDCs and possibly by other immune cells after HIV-1 infection [21,22,24]. When we compared Siglec-1 expression levels on distinct myeloid cells activated in the presence of IFNα, we observed a 17-fold up-regulation in monocyte-derived DCs and a twofold up-regulation in macrophages and monocytes (Figure 1A).

We have previously reported that Siglec-1 expression levels determine the capacity of DCs to capture HIV-1 [9]. To test whether this also holds true for monocytes and macrophages, we first compared the density of Siglec-1 surface expression applying a quantitative FACS assay that determines the absolute number of Siglec-1 antibody binding sites. This number was highest in IFNα-activated monocyte-derived DCs, followed by macrophages and monocytes (Figure 1B). Next, we analyzed binding of infectious HIV-1 to IFNα-activated myeloid cells. HIV-1 was incubated with the cells for 4 h at 4°C to avoid viral internalization and cell-associated p24^Gag^ was quantified by ELISA after extensive washing. Consistent with their respective Siglec-1 expression levels (Figure 1B), IFNα-activated monocyte-derived DCs showed a higher HIV-1 binding capacity than monocytes (Figure 1C). To investigate whether this binding was specific for Siglec-1, cells were pre-treated with a monoclonal antibody (mAb) against Siglec-1. This treatment led to a reduction of HIV-1 binding by 83% in all cases, while isotype control treatment had no inhibitory effect (Figure 1D). Similar results were obtained with fluorescent HIV-1 Virus-Like Particles (VLPs), which lack the viral envelope glycoprotein but carry sialyllactose-containing gangliosides recognized by Siglec-1 (Additional file 1: Figure S1A-B). These data indicate that Siglec-1 is the main molecule responsible for HIV-1 capture by IFNα-activated myeloid cells, and that its expression correlates with the viral binding capacity of the respective cell type.

### Siglec-1 mediates HIV-1 uptake into a storage compartment and enhances HIV-1 *trans*-infection specially in IFNα-treated monocytes and DCs

**Figure 2:**
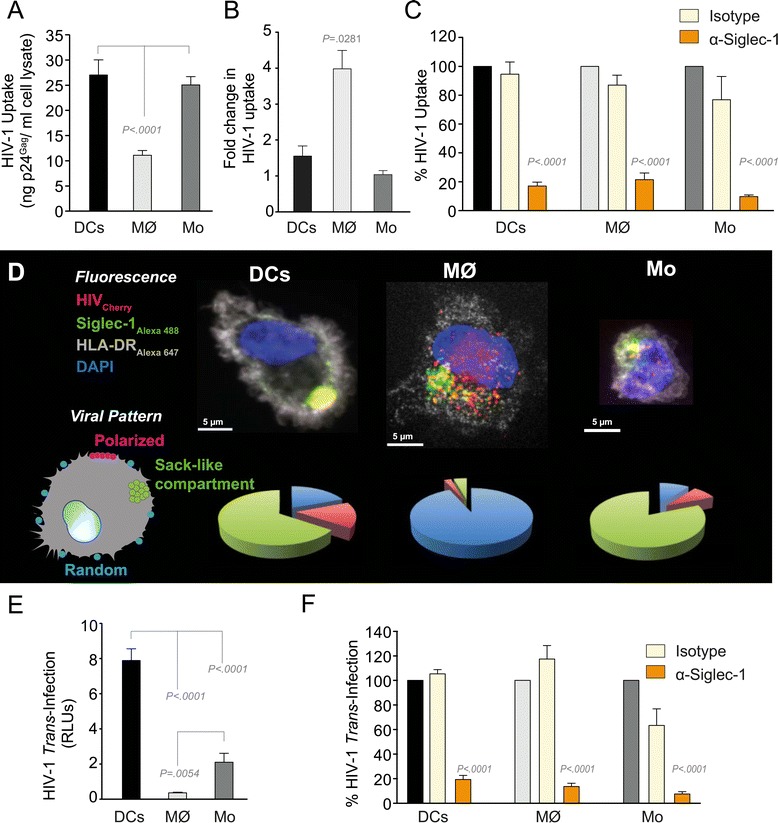
Siglec-1 mediates HIV-1 uptake into a storage compartment and enhances HIV-1 *trans*-infection specially in IFNα-treated monocytes and DCs. **A**. Uptake of HIV-1_NL4–3_ by different myeloid cells exposed to IFNα. Cells were cultured with HIV-1 to measure p24^Gag^ by ELISA. Mean values and SEM from four experiments include cells from 12 donors. **B**. Fold change in HIV-1_NL4–3_ uptake of cells treated with bafilomycin A1 compared to untreated cells. Mean values and SEM include cells from three donors. **C**. Relative uptake of HIV-1_NL4–3_ by IFNα-treated myeloid cells pre-incubated with the indicated mAbs. Values are normalized to the level of HIV-1 uptake by mock-treated cells (set at 100%). Mean values and SEM from two experiments include cells from six donors. **D.** Confocal microscopy analysis of different IFNα-treated myeloid cells pulsed with HIV-1_Cherry_ and stained for Siglec-1 (Alexa 488), HLA-DR (Alexa 647) and DAPI. (Top) Representative viral pattern for each kind of myeloid cell analyzed, showing maximum fluorescence intensity of four channels. (Bottom) Percentage of myeloid cells with distinct viral patterns: random distribution, polarized accumulation, and sac-like compartment formation, as illustrated in the left drawing. Mean values of 50 cells from two different donors are shown. **E.** HIV-1 transmission from IFNα-treated myeloid cells to a luciferase reporter CD4^+^ cell line. HIV-1 infection was determined by induced luciferase activity in relative light units (RLUs). Mean values and SEM from four experiments include cells from 12 donors. **F.** Relative HIV-1 transmission from IFNα-treated myeloid cells pre-incubated with the indicated mAbs. Values are normalized to the level of HIV-1 *trans*-infected by mock-treated cells. Mean values and SEM from two experiments include cells from six donors. Statistical differences were assessed with a paired t test in **A** and **E**, and with a one sample t-test in **B**, **C** and **F**.

Having established Siglec-1-dependent virus binding in all three types of myeloid cells, we performed uptake experiments at 37°C to follow the fate of the bound virus. IFNα-activated myeloid cells were incubated with HIV-1 for 4 h at 37°C and cell-associated p24^Gag^ was quantified by ELISA after extensive washing (Figure 2A). Monocyte-derived DCs and monocytes contained similar amounts of HIV-1, while macrophages displayed lower uptake (Figure 2A). Treatment with Bafilomycin A1, an inhibitor of lysosomic degradation, only increased the level of cell-associated virus in macrophages (Figure 2B). Thus, faster viral degradation in macrophages accounts for the reduced cell-associated virus observed in this cell type (Figure 2A). HIV-1 uptake was strongly inhibited by a mAb against Siglec-1 in all cases (Figure 2C). Similar results were obtained when we performed uptake experiments with fluorescent VLPs (Additional file 1: Figure S1C-D), with macrophages showing residual capture in the presence of the mAb (Additional file 1: Figure S1D).

To elucidate the HIV-1 trafficking differences in macrophages compared to other myeloid cells, we investigated viral uptake by confocal microscopy. IFNα-treated myeloid cells were pulsed with fluorescent HIV-1_Cherry_ for 4 h at 37°C and subsequently stained with mAbs against Siglec-1 and against HLA-DR to reveal cellular membranes (Figure 2D, top images). While most of the monocyte-derived DCs and monocytes accumulated HIV-1_Cherry_ within a sac-like compartment enriched in Siglec-1, macrophages exhibited a more scattered pattern for cell-associated HIV-1_Cherry_ (Figure 2D). Thus, the complementary approaches of confocal microscopy analysis and viral uptake experiments indicate that Siglec-1 is essential for HIV-1 capture in all myeloid cells, while it is not sufficient for further downstream uptake and trafficking involved in the formation of a condensed viral compartment.

We next assessed the capacity for HIV-1 *trans*-infection by the different myeloid cell types. Cells were pulsed with equal amounts of the X4-tropic HIV-1_NL4–3_ followed by extensive washing and were subsequently co-cultured with a CD4^+^ reporter cell line for two days. Monocyte-derived DCs had the highest capacity for *trans*-infection followed by monocytes, while macrophages showed only weak *trans*-infection capacity (Figure 2E), consistent with their faster viral degradation kinetics (Figure 2B). However, *trans*-infection depended on Siglec-1 in all cases (Figure 2F).

### Siglec-1 is up-regulated on monocytes from HIV-1-infected individuals, and its expression is reduced upon successful antiretroviral treatment

**Figure 3:**
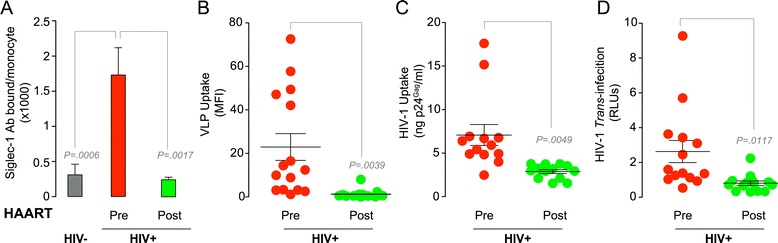
Siglec-1 is up-regulated on monocytes of HIV-1-infected individuals, but its expression is reduced after successful antiretroviral treatment. **A**. Mean number of Siglec-1 Ab binding sites per cell displayed by monocytes isolated from HIV-1-negative men and from HIV-1-infected men before or after successful antiretroviral treatment (with an initial median of 297 CD4^+^ T-cell count and 5 log_10_ HIV RNA copies/ml that changed to a median of 505 CD4^+^ T-cell count and 1.7 log_10_ HIV RNA copies/ml after treatment). Graph shows mean values and SEM from nine HIV-1-negative individuals and 16 HIV-1-infected individuals. Man Whitney t-test was used to compare differences between HIV-1-negative individuals and HIV-1-infected individuals. Paired t-test was used to assess differences between HIV-1-infected men before or after successful antiretroviral treatment. **B**. Uptake of fluorescent VLPs by monocytes isolated from HIV-1-infected individuals before (red dots) and after (green dots) antiretroviral treatment. Cells were pulsed with VLPs for 3 h at 37°C and assessed by FACS. Graph shows mean values and SEM from 16 HIV-1-infected individuals. **C**. Uptake of HIV-1_NL4–3_ by monocytes isolated from HIV-1-infected individuals before and after antiretroviral treatment. Cells were cultured with HIV-1 for 4 h at 37°C, washed and lysed to measure p24^Gag^ by an ELISA. Graph shows mean values and SEM of the 13 individuals from which we recovered enough monocytes to perform this assay. **D.** HIV-1 transmission from monocytes isolated from HIV-1-infected individuals before and after antiretroviral treatment to a reporter CD4^+^ cell line cultured at a ratio 5:1. Cells were pulsed with HIV-1_NL4–3_ as in Figure 2D. Graph shows mean values and SEM from the same individuals of panel **C**.

To explore whether Siglec-1 could be functionally important *in vivo,* we assessed Siglec-1 expression on blood monocytes from HIV-1-infected individuals before and after initiation of antiretroviral treatment (Table 1), and compared them to HIV-1-negative individuals. When we quantified the number of Siglec-1 Ab binding sites per monocyte *ex vivo* we found that Siglec-1 expression was significantly lower in HIV-1-negative individuals compared to untreated HIV-1-infected individuals (Figure 3A; 6-fold difference, *P* = 0.0006). Furthermore, Siglec-1 expression was significantly higher in monocytes isolated before antiretroviral treatment compared to monocytes isolated after antiretroviral treatment from the same patients (Figure 3A; 7-fold difference, *P* = 0.0017), returning to the levels showed by HIV-1-negative individuals. Next, we compared monocytes from HIV-1-infected individuals for their capacity to take up VLPs. Consistent with their higher expression of Siglec-1, cells isolated before antiretroviral treatment exhibited a higher uptake capacity for VLPs compared to cells obtained under suppressive therapy (Figure 3B; 19-fold difference, *P* = 0.0039). Similar results were observed for uptake of complete HIV-1 (Figure 3C). Consistent with the enhanced uptake, HIV-1 *trans*-infection was also higher for cells taken before antiretroviral treatment (Figure 3D, *P* = 0.0117). These results indicate that *in vivo,* Siglec-1 expression on peripheral blood monocytes is up-regulated by HIV-1 infection, but normalizes after effective antiretroviral treatment suppresses viral replication and the associated immune activation [17,28].

**Table 1:** **Characteristics of the HIV-1-infected men followed longitudinally before and after initiation of antiretroviral treatment that are shown in Figures** 3 **and** 4

**PRE/POST Antiretroviral treatment**	**Number or Median (IQR)**
Number of patients	16
Age (years)	36 (24–40)
Antiretroviral treatment regimen	
*Non-Nucleoside Reverse Transcriptase Inhibitors*	8
*Protease Inhibitors*	7
*Integrase Inhibitors*	1
Time from diagnosis to antiretroviral treatment (months)	2 (1.5-5)
Time between samples (months)	11 (7–19)
CD4^+^ T-cell count (cells/μl)	
*PRE antiretroviral treatment*	297 (239–316)
*POST antiretroviral treatment*	505 (401–597)
Plasma Viral Load (Log_10_ HIV RNA copies/ml)	
*PRE antiretroviral treatment*	5 (4.4-5.5)
*POST antiretroviral treatment*	1.7 (1.4-1.7)

### The plasma of untreated HIV-1-infected individuals stimulates Siglec-1 expression and signals via the type I IFN receptor

**Figure 4:**
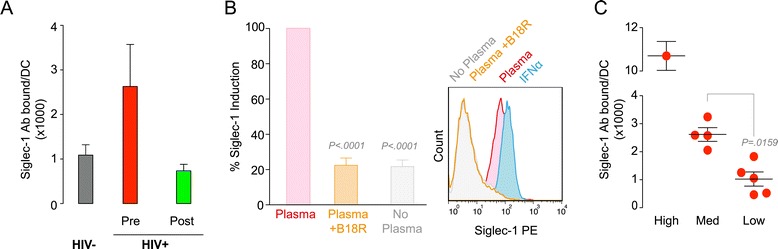
The plasma of untreated HIV-1-infected individuals stimulates Siglec-1 expression and signals via type I IFN receptor. **A.** Mean number of Siglec-1 Ab binding sites per cell induced by the plasma of HIV-1-negative individuals and HIV-1-infected individuals before or after successful antiretroviral treatment, respectively. DCs derived from uninfected donors were cultured for 24 h in the presence of plasma and then stained for Siglec-1. Graph shows mean values and SEM of Siglec-1 induction in DCs from two donors that were tested in parallel with the plasmas from five HIV-1-negative individuals and ten HIV-1-infected individuals. **B**. Relative blockade of Siglec-1 expression by B18R, a soluble recombinant receptor with high affinity for type I IFNs, which inhibits Siglec-1 induction triggered by the plasmas of untreated HIV-1-infected individuals. DCs were cultured for 24 h with the respective plasma in the presence or absence of 2 μg/ml of B18R. Values are normalized to the level of Siglec-1 induction by plasma of mock-treated cells (set at 100%). Mean changes from 100% were assessed with a one sample t-test. Representative histogram also depicts IFNα-treated DCs. **C.** Mean number of Siglec-1 Ab binding sites per cell induced by the plasma of untreated HIV-1-infected individuals. DCs were cultured for 24 h in the presence of plasma collected from patients displaying the highest levels of Siglec-1 (>5500 Ab binding sites per monocyte), intermediate levels of Siglec-1 (4000–2500 Ab binding sites per monocyte) or the lowest levels of Siglec-1 (<1500 Ab binding sites per monocyte) and then stained for Siglec-1. Graph shows mean values and SEM of Siglec-1 induction in DCs from two donors that were tested in parallel with the plasmas from ten HIV-1-infected individuals. Man Whitney t-test was used to compare the differences between distinct plasmas to induce Siglec-1 expression.

To assess if immune activating factors present in the plasma could trigger Siglec-1 expression on myeloid cells, we tested the capacity of such plasma to induce Siglec-1 expression on DCs derived from uninfected donors. When we quantified the number of Siglec-1 Ab binding sites per DC, we found that plasma from untreated HIV-1-infected individuals triggered Siglec-1 expression to a higher extent than plasma from HIV-1-negative individuals (Figure 4A). Induction of Siglec-1 expression was reduced to the level triggered by plasma from uninfected individuals when plasma from the same HIV-1-infected individuals but isolated after antiretroviral treatment was used (Figure 4A). This effect was mediated by signaling through the type I IFN receptor, since B18R, a soluble recombinant receptor with high affinity for type I IFNs, blocked Siglec-1 induction triggered by plasma from untreated HIV-1-infected patients (Figure 4B). Furthermore, addition of IFNα up-regulated Siglec-1 expression to similar levels as the plasma from untreated HIV-1-infected patients (Figure 4B). Moreover, plasma from those untreated HIV-1-infected individuals that displayed the highest level of Siglec-1 Ab binding sites per monocyte in peripheral blood was able to trigger Siglec-1 expression in donor DCs to a higher extent than plasma from individuals exhibiting lower levels of Siglec-1 (Figure 4C). Thus, the capacity to induce Siglec-1 via soluble factors in the plasma of HIV-1-infected individuals is related to Siglec-1 levels on the surface of monocytes from the respective donor, indicating that Siglec-1 expression *in vivo* is indeed regulated by soluble activation factors signaling via the type I IFN receptor.

### Expression of Siglec-1 on monocytes correlates with clinical parameters

**Figure 5:**
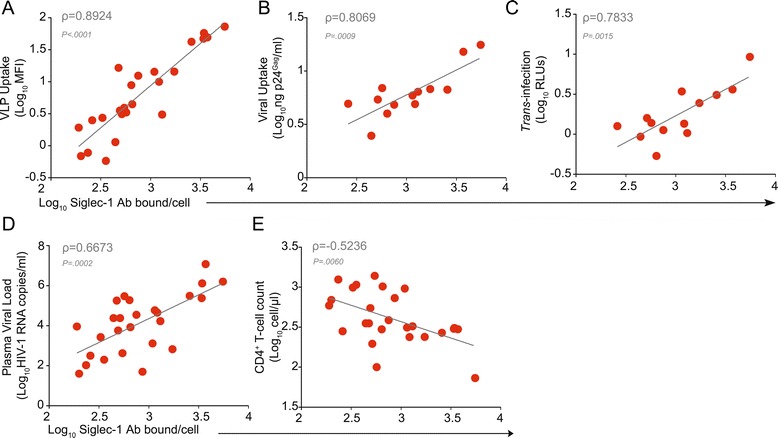
Expression of Siglec-1 on monocytes correlates with clinical parameters. **A.** Positive correlation between the mean number of Siglec-1 Ab binding sites per cell and VLP uptake of monocytes isolated from antiretroviral treatment-naïve HIV-1-infected men, with a median of 338 CD4^+^ T-cell count and 4.3 log_10_ HIV RNA copies/ml. Graph shows the linear correlation of 26 individuals. Pearson correlation coefficient of the population is denoted by ρ. **B.** Positive correlation between the mean number of Siglec-1 Ab binding sites per cell and HIV-1 uptake of monocytes isolated from antiretroviral treatment-naïve HIV-1-infected individuals. Graph shows the linear correlation for the 13 individuals from which we recovered enough monocytes to perform this assay. **C.** Positive correlation between the mean number of Siglec-1 Ab binding sites per cell and the HIV-1 *trans*-infection capacity of monocytes isolated from antiretroviral treatment-naïve HIV-1-infected individuals. Graph shows the linear correlation for the same individuals as in panel **B. D.** Positive correlation between the mean number of Siglec-1 Ab binding sites per cell and the plasma viral load at the time of sample analysis of monocytes isolated from antiretroviral treatment-naïve HIV-1-infected individuals. Graph shows a linear correlation for 26 individuals. **E.** Negative correlation between the mean number of Siglec-1 Ab binding sites per cell and the CD4^+^ T cell count at the time of sample analysis of monocytes isolated from antiretroviral treatment-naïve HIV-1-infected individuals. Graph shows a linear correlation for 26 individuals.

Focusing our analysis on antiretroviral treatment-naïve patients (Table 2), we found a positive correlation between Siglec-1 expression levels on isolated monocytes and i) VLP uptake (Figure 5A; ρ = 0.8924; *P* < 0.0001), ii) HIV-1 uptake (Figure 5B; ρ = 0.8069; *P* = 0.0009), and iii) HIV-1 *trans*-infection capacity (Figure 5C; ρ = 0.7833; *P* = 0.0015). In addition, Siglec-1 expression levels positively correlated with plasma viral load (Figure 5D; ρ = 0.6673; *P* = 0.0002). Conversely, Siglec-1 expression negatively correlated with CD4^+^ T-cell counts (Figure 5E; ρ = −0.5236; *P* = 0.006).

**Table 2:** **Characteristics of the HIV-1-infected men before initiation of antiretroviral treatment, whose samples were used in Figure** 5

**PRE Antiretroviral treatment**	**Number or Median (IQR)**
Number of patients	26
Age (years)	36 (24–40)
CD4^+^ T-cell count (cells/μl)	338.5 (285–721)
Plasma Viral Load (Log_10_ HIV RNA copies/ml)	4.3 (2.9-5.3)

### Siglec-1 positive cells accumulate in inflamed lymphoid tissues in areas enriched in CD4^+^ T cells

**Figure 6:**
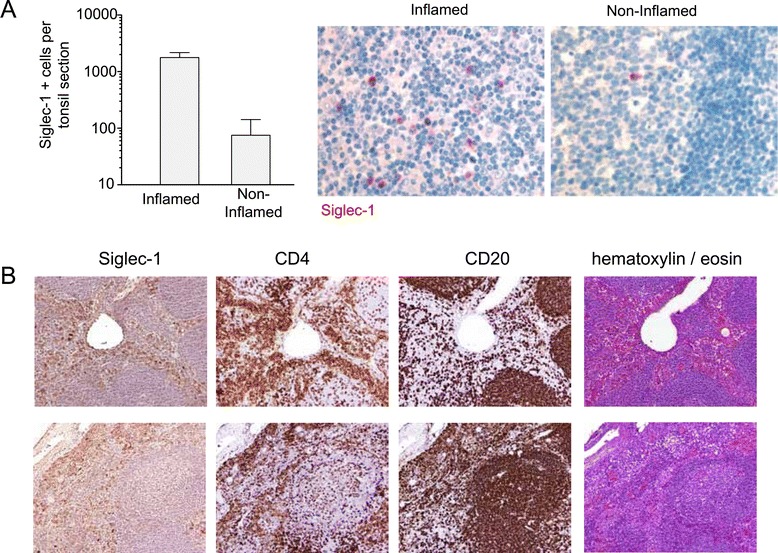
Siglec-1 positive cells accumulate in inflamed lymphoid tissues in areas enriched in CD4^+^ T cells*.* **A.** Siglec-1 expression on sections of tonsils surgically removed either due to acute tonsillitis (n = 3; inflamed) or obstructive hypertrophy (n = 3; non-inflamed). Graph show mean values and SEM. Representative staining images are depicted in the right panels (magnification 40x). **B.** Staining images of Siglec-1 on lymph nodes surgically removed from an HIV-1-infected individual under antiretroviral treatment. Four continuous sections were cut and labeled independently with the following panel: anti-Siglec-1, anti-CD4, anti-CD20 or hematoxilin/eosin. Top images show perivascular distribution of Siglec-1 positive cells and bottom images display a perifollicular and subcapsular distribution.

To establish whether Siglec-1 can be detected in lymphoid tissues where myeloid cells establish continuous cell-to-cell interactions that could favor viral transmission, we first performed immunohistochemical analyses of tissues from HIV-1 uninfected individuals. Sections from paraffin-embedded tonsils derived from HIV-, HBV- and HCV-negative patients were classified as inflamed (n = 3) or non-inflamed (n = 3) based on histopathological criteria. Inflamed tonsils harbored on average 23-fold more Siglec-1-positive cells than non-inflamed tonsils (Figure 6A), indicating a clear association between the degree of immune activation and the number of cells expressing the *trans*-infection receptor.

We next assessed whether Siglec-1 expressing cells could be detected in lymphoid tissue from an HIV-1-infected individual who had lymph nodes removed before and after initiation of antiretroviral treatment. Of note, this patient also had an untreated HCV infection. Siglec-1 positive cells were detected in perivascular, sub-capsular, and perifollicular areas enriched in CD4^+^ T cells, but mostly excluded from CD20-positive follicular zones (Figure 6B). A similar pattern was observed for tissue obtained before and after initiation of therapy. Although we cannot rule out the possibility that untreated HCV infection sustained Siglec-1 expression, it is also conceivable that antiretroviral drug concentrations were insufficient to fully suppress HIV-1 replication in the lymphoid tissue of this particular individual [29,30] thus sustaining IFNα production.

### Siglec-1 mediates HIV-1 *trans*-infection by myeloid cells isolated from lymphoid tissue

**Figure 7:**
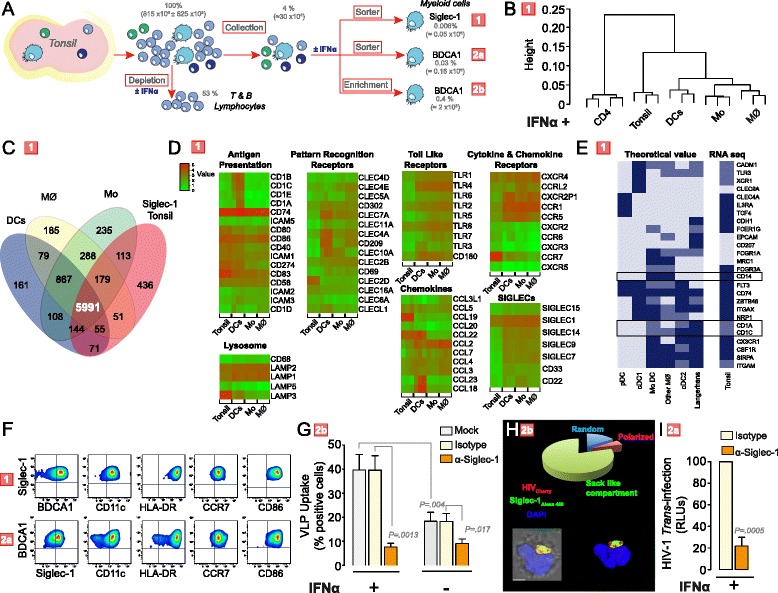
Siglec-1 mediates HIV-1 trans-infection in myeloid cells isolated from lymphoid tissue. **A.** Workflow to enrich or isolate tonsillar myeloid cells. Red numbers on the right are used throughout the figure to identify isolation procedure. **B.** Hierarchical clustering (by Spearman correlation) of IFNα-treated sorted Siglec-1 positive tonsillar cells and primary cells based on protein-coding gene expression. **C.** Venn diagram of overlapping protein-coding genes in the indicated IFNα-treated cells. **D.** Expression heat maps of genes with antigen-presenting cell functions shared between IFNα-treated Siglec-1 sorted tonsillar cells and at least one myeloid cell type. Data in **B**-**D** show three donors for each cell type. **E.** Heat map-like representation of the theoretical presence/absence profile reported for markers proposed for myeloid cell classification [31] in the indicated cellular subsets (dark blue: presence; light blue: absence; blue: heterogeneous expression/unknown/unclear). Observed expression in sorted Siglec-1 tonsillar cells is also depicted (dark blue: >1.5; light blue: <1; blue: ≥1 and ≤1.5 log_10_). Boxes indicate markers of inflammatory myeloid cells [32]. **F.** Representative FACS staining of selected myeloid markers in sorted Siglec-1 positive cells (top) and BDCA1-positive cells (bottom). **G.** Uptake of VLPs by BDCA1-positive tonsillar cells exposed to IFNα or left untreated. Cells were pre-incubated with indicated mAbs. Mean values and SEM from three experiments include cells from eight donors, assessed with a paired t-test. **H.** Confocal microscopy of IFNα-treated BDCA1-positive tonsillar cells (n = 2) pulsed with HIV-1_Cherry_ and stained for Siglec-1. Representative viral pattern (maximum fluorescence intensity of three channels and bright field). **I.** HIV-1 transmission from IFNα-treated sorted BDCA1-positive tonsillar cells to a reporter CD4^+^ cell line. Cells were pre-incubated with indicated mAbs. Values are normalized to the level of HIV-1 *trans*-infected by isotype-treated cells. Mean values and SEM from two experiments include mixed cells from six donors, assessed with a one sample t-test.

Detection of Siglec-1 on cells residing in lymphoid tissues prompted us to further characterize the role of tonsil-derived Siglec-1 positive cells in HIV-1 capture and *trans*-infection *ex vivo*. Cells were isolated from non-inflamed tonsils of HIV-1-uninfected individuals as depicted in Figure 7A. After mechanical disruption, mononuclear tonsillar cells were isolated by Ficoll-Hypaque gradient centrifugation. T- and B-lymphocytes were subsequently depleted with magnetic beads and the remaining cell fraction was cultured in the presence of IFNα or left untreated. FACS analysis revealed an up-regulation of Siglec-1 after 24 h of IFNα treatment.

We next isolated Siglec-1 positive cells by sorting of IFNα-treated tonsillar cells and performed a full transcriptome RNA-seq analysis. Results were compared to a similar RNA-seq analysis of the previous IFNα-treated myeloid cells (DCs, macrophages and monocytes), and also to CD4^+^ T cells exposed to IFNα. Hierarchical clustering of samples on the basis of their protein coding gene expression levels revealed that sorted Siglec-1 positive tonsillar cells clustered closer to the other myeloid cells and away from CD4^+^ T cells (Figure 7B). Sorted Siglec-1 positive cells shared almost 6000 protein coding genes with other myeloid cells (expressed more than 100 library size-normalized reads per kilobase of exonic sequence, Figure 7C), among which we found a significant enrichment of genes related to antigen-presenting functions (Table 3). Analysis of transcript levels of genes related to antigen-presenting functions revealed heterogeneous gene expression levels across individual myeloid cells and tonsil-derived Siglec-1 positive cells (Figure 7D). Siglec-1 was highly expressed in all cell types (more than 2,000 library size-normalized reads per kilobase of exonic sequence). Specific markers of tissue origin, such as CCR7 and CCL19, were found overexpressed in tonsil-derived Siglec-1 positive cells (Figure 7D). When we plotted a heat map-like representation based on a panel of markers recently proposed for identifying different human mononuclear phagocyte subsets [31] tonsil-derived Siglec-1 positive cells expressed most of those markers, but showed a distinctive profile (Figure 7E). Siglec-1 positive tonsillar cells expressed CD1c (BDCA1), CD1a and CD14 (Figure 7E), which are all markers found in other primary human myeloid cells isolated from inflammatory fluids [32]. Thus, the unique pattern of tonsil-derived Siglec-1 positive cells might reflect the complexity of classifying mononuclear phagocytes under inflammatory conditions [31]. Overall, transcriptomic analysis indicated that sorted Siglec-1 tonsillar cells presented a unique myeloid antigen-presenting cell profile.

**Table 3:** **List of GO biological processes relevant for antigen-presenting cell function found significantly enriched after Bonferroni correction in the 5991 protein coding genes commonly expressed by sorted tonsil-derived Siglec-1 positive cells and the different types of myeloid cells exposed to IFNα**

***GO biological process***	***Genes in GO process***	***Genes found***	***Expected***	***P-value***
**Immune system process (GO:0002376)**	1875	791	5,10E + 05	1,38E-27
Regulation of immune system process (GO:0002682)	1090	439	2,96E + 05	3,63E-09
Positive regulation of immune system process (GO:0002684)	636	273	1,73E + 05	2,06E-06
Defense response (GO:0006952)	1256	490	3,42E + 05	1,30E-08
Regulation of defense response (GO:0031347)	502	218	1,37E + 05	1,69E-04
Immune response (GO:0006955)	1153	476	3,14E + 05	3,06E-12
Regulation of immune response (GO:0050776)	712	303	1,94E + 05	2,89E-07
Positive regulation of immune response (GO:0050778)	428	200	1,16E + 05	2,52E-06
Activation of immune response (GO:0002253)	344	175	9,35E + 04	7,68E-08
Immune response-activating signal transduction (GO:0002757)	296	165	8,05E + 04	2,15E-10
Immune response-regulating signaling pathway (GO:0002764)	403	202	1,10E + 05	3,18E-09
Immune effector process (GO:0002252)	399	194	1,09E + 05	1,82E-07
Response to virus (GO:0009615)	250	129	6,80E + 04	7,85E-05
**Innate immune response (GO:0045087)**	773	362	2,10E + 05	6,72E-16
Regulation of innate immune response (GO:0045088)	246	141	6,69E + 04	4,76E-09
Positive regulation of innate immune response (GO:0045089)	178	106	4,84E + 04	1,63E-06
Activation of innate immune response (GO:0002218)	148	94	4,02E + 04	1,04E-06
Innate immune response-activating signal transduction (GO:0002758)	141	91	3,83E + 04	1,05E-06
**Antigen processing and presentation (GO:0019882)**	222	142	6,04E + 04	5,93E-13
Of peptide antigen (GO:0048002)	186	126	5,06E + 04	8,41E-13
Of peptide antigen via MHC class I (GO:0002474)	101	81	2,75E + 04	3,02E-10
Of exogenous antigen (GO:0019884)	172	119	4,68E + 04	1,77E-12
Of exogenous peptide antigen (GO:0002478)	170	117	4,62E + 04	5,08E-12
Of exogenous peptide antigen via MHC class I (GO:0042590)	79	64	2,15E + 04	3,07E-07
Of exogenous peptide antigen via MHC class I, TAP-dependent (GO:0002479)	75	61	2,04E + 04	9,76E-07
**Pattern recognition receptor signaling pathway (GO:0002221)**	138	89	3,75E + 04	1,97E-06
**Toll-like receptor signaling pathway (GO:0002224)**	120	80	3,26E + 04	5,80E-06
Toll-like receptor 3 signaling pathway (GO:0034138)	79	57	2,15E + 04	5,06E-04
Toll-like receptor 4 signaling pathway (GO:0034142)	95	63	2,58E + 04	1,52E-03
Toll-like receptor 9 signaling pathway (GO:0034162)	73	52	1,99E + 04	4,80E-03
TRIF-dependent toll-like receptor signaling pathway (GO:0035666)	76	54	2,07E + 04	2,56E-03
**Cellular response to cytokine stimulus (GO:0071345)**	457	218	1,24E + 05	3,25E-08
**Cytokine-mediated signaling pathway (GO:0019221)**	339	169	9,22E + 04	1,08E-06
**Regulation of type I interferon production (GO:0032479)**	106	78	2,88E + 04	9,02E-08
Positive regulation of type I interferon production (GO:0032481)	75	56	2,04E + 04	2,13E-04

After GO enrichment analysis, 507 GO processes were found significantly enriched, where most of the genes (3564) were related to cellular metabolic processes (GO:0044237). Here we just summarize the enriched GO processes related to immune function, the number of genes categorized in each GO function, the actual number of genes found in the 5991 protein coding genes commonly expressed, the expected *P* value and the real *P* value obtained for the genes of interest.

Sorted Siglec-1 positive cells from IFNα-treated tonsils co-stained with several myeloid markers that had been identified in the transcriptomic analysis, including BDCA1, CD11c, HLA-DR, CCR7 and CD86 (Figure 7F, top panels). However, sorted Siglec-1 positive cells could not be employed in functional assays, since mAbs against Siglec-1 block HIV-1 capture (Figure 1D). When we sorted BDCA1-positive cells from IFNα-treated tonsillar cells, they also stained positive for Siglec-1, CD11c, HLA-DR, CCR7 and CD86 (Figure 7F, bottom panels), indicating that this population had a comparable phenotype to that exhibited by Siglec-1 positive cells and could be used for functional assays.

Viral uptake experiments performed with IFNα-treated BDCA1-positive tonsillar cells demonstrated a higher VLP capture capacity when compared to mock-treated BDCA1-positive cells (Figure 7G), and was specifically inhibited by pre-treatment with an anti-Siglec-1 mAb (Figure 7G). Of note, neither the BDCA1-negative cell population nor B cells, which express BDCA1 and could thus be present in the BDCA1-positive cell fraction, were able to up-regulate VLP uptake after IFNα treatment (Additional file 1: Figure S2). In order to investigate HIV-1 trafficking in IFNα-treated BDCA1-positive cells, we added fluorescent HIV-1_Cherry_ for 4 h at 37°C and subsequently stained cells with an anti-Siglec-1 mAb (Figure 7H). Confocal microscopy indicated that most of these BDCA1-positive cells accumulated HIV-1_Cherry_ within a sac-like compartment enriched in Siglec-1, as previously observed for DCs and monocytes (Figure 2C).

Finally, to work with highly purified cell populations, we sorted BDCA1^+^CD2^−^CD20^−^-tonsillar cells cultured in the presence of IFNα and assessed Siglec-1 involvement in HIV-1 *trans*-infection. IFNα-activated BDCA1-positive cells pre-treated with isotype control or specific mAb were exposed to HIV-1 for 4 h at 37°C, extensively washed and co-cultured with a CD4^+^ reporter cell line for 2 days (Figure 7I). T*rans*-infection was readily observed and was specifically inhibited by pre-treatment with a mAb against Siglec-1 (Figure 7I). These results indicated that *ex vivo*, activation of myeloid cells from tonsils with IFNα leads to Siglec1-dependent enhanced HIV-1 capture and *trans*-infection, supporting a potential role of Siglec-1 as an important molecule that could contribute to viral capture and *trans*-infection within lymphoid tissues in HIV-1-infected individuals.

## Discussion

In this report, we show that Siglec-1 on myeloid cells (i) is up-regulated by IFNα; (ii) mediates HIV-1 capture and *trans*-infection; (iii) correlates *in vivo* with the levels of plasma viral load and diminishes after effective antiretroviral treatment, and (iv) is expressed in lymphoid tissues in an inflammation-dependent manner where it can mediate HIV-1 *trans*-infection. Taken together, these findings indicate that inflammatory processes or immune activating signals triggered by HIV-1 replication, such as IFNα release, stimulate Siglec-1 expression on myeloid cells, a process that could enhance viral capture and *trans*-infection of CD4^+^ target T cells within lymphoid tissues. Based on our results, this mechanism may be driven by IFNα-activated monocytes and DCs, which exhibited higher Siglec-1 dependent *trans*-infection than macrophages. Yet, despite the faster viral degradation of captured virions in macrophages, Siglec-1 expression in this cell type may facilitate productive HIV-1 *cis*-infection [33].

This model is consistent with our findings that *in vivo* Siglec-1 expression is up-regulated on monocytes from HIV-1-infected individuals, but diminishes after effective antiretroviral treatment suppresses plasma viral load and virus-induced activating signals [17,28]. Our results are in line with previous reports showing Siglec-1 up-regulation on circulating monocytes of HIV-1-infected individuals with higher plasma viral loads [18,34]. However, assays performed here provide functional evidence that monocytes isolated directly from HIV-1-infected individuals capture HIV-1 and *trans*-infect CD4^+^ target cells. We also found that Siglec-1 expression increased with plasma viral load and decreased with CD4^+^ T-cell counts in HIV-1 infected patients. Furthermore, stimuli present in the plasma of untreated HIV-1-infected individuals induced Siglec-1 expression on myeloid cells via type I IFN receptor signaling. Overall, these data suggest that Siglec-1 could become a useful biomarker to monitor chronic immune activation driven by HIV-1 infection.

Detection of Siglec-1 within lymphoid tissues suggests that this receptor could mediate HIV-1 capture and transmission in these compartments. Lymphoid tissues are the perfect scenarios to fuel novel infections, since they are major sites of HIV-1 replication [35], where plasmacytoid and myeloid cells accumulate during the course of HIV-1 infection [25,26] and IFNα is detected in lymph nodes of HIV-1-infected individuals [24]. Functional assays performed here with myeloid cells isolated from tonsils and activated with IFNα (to mimic the immune activation state driven by HIV-1 infection in the lymphoid tissues), identified Siglec-1 as a key receptor involved in viral capture and transmission.

## Conclusions

We have shown that Siglec-1 expression on distinct primary myeloid cells can be induced during chronic HIV-1 infection *in vivo* and contribute to systemic viral dissemination. Our results strongly support that Siglec-1 is an important molecule that could accelerate HIV-1 transmission in the crowded cellular environment of lymphatic tissues, where many T-cells can contact myeloid cells. Future studies aimed at blocking Siglec-1 in adequate animal models will be required to shed light on the relative contribution of HIV-1 *trans*-infection to disease progression and might offer novel therapeutic approaches to halt HIV-1 cell-to-cell transmission.

## Methods

### Ethics statement

The institutional review board on biomedical research from Hospital Germans Trias i Pujol (HUGTIP) approved this study. All patients involved in this study gave their written informed consent to participate.

### Primary cells

Peripheral blood mononuclear cells (PBMCs) were obtained from HIV-1-seronegative donors by Ficoll-Hypaque density gradient centrifugation and monocyte populations were isolated as described in [36]. Monocytes were differentiated into DCs with 1000 U/ml of granulocyte-macrophage colony-stimulating factor plus 1000 U/ml of Interleukin-4 (both from R&D). In parallel, monocytes from the same donors were differentiated into macrophages with 100 ng/ml of macrophage colony-stimulating factor (Preprotech). Cells were cultured for 7 days, and cytokines and media were replaced every two days. At day five, monocytes, DCs and macrophages were stimulated with 1000 U/ml of Interferon-2α (Sigma-Aldrich) for two days.

HIV-1-infected individuals were selected from a cohort of patients with samples collected before and after antiretroviral treatment. Patient’s characteristics are described in Table 1. HIV-1-negative males matched for age were included as healthy controls. To perform functional assays, PBMCs were thawed and monocytes were isolated with CD14^+^ magnetic beads (Miltenyi Biotec). Of note, positive isolation did not up-regulate Siglec-1 expression.

Human tonsils were removed during tonsillectomies of individuals undergoing prescribed surgery at the HUGTIP. After mechanical disruption, mononuclear tonsillar cells were isolated by Ficoll-Hypaque gradient centrifugation. T- and B-lymphocytes were subsequently depleted with magnetic beads against CD3^+^ and CD19^+^ (Miltenyi Biotec) prior blocking of the Fc receptor (Miltenyi Biotec). Siglec-1 positive cells were sorted by isolating Siglec-1^+^/CD20^−^/CD2^−^cells with mAbs7–239 α-Siglec-1-PE (AbD Serotec), α-CD2-PerCP and α-CD20-PerCP (both from Becton Dickinson) in a FACSVantage SE after blocking Fc receptors with 1 mg/ml of human IgGs (hIgGs; Privigen, Behring CSL). Myeloid cells were also isolated either by BDCA1 positive selection with magnetic beads (Miltenyi Biotec) or by sorting BDCA1^+^/CD20^−^/CD2^−^cells using mAb α-BDCA1-PE (Miltenyi Biotec), α-CD2-PerCP and α-CD20-PerCP. Cells were stimulated with 1000 U/ml IFNα for 24–48 h. Isolated cells were blocked with 1 mg/ml of hIgGs for 20 min at RT and stained with the following mAbs: α-CD11c-APC-Cy7 (BioLegend), α-CCR7-PerCP (Biolegend), α-Siglec-1-Alexa 488 (AbD Serotec), α-HLA-DR-V450 (BD), α-CD86-FITC (BD), α-BDCA1-PECy7 (Biolegend), α-CD20-PerCPCy5.5 (BD), α-CD3-PerCP (BD) at 4°C for 30 min. Samples were analyzed with LSRII using FlowJo software to evaluate collected data.

All primary cells were cultured in RPMI containing 10% fetal bovine serum (FBS), 100 U/ml of penicillin and 100 μg/ml of streptomycin (all from Invitrogen).

### Siglec-1 surface expression analysis by FACS

2x10^5^ myeloid cells were blocked with 1 mg/ml of hIgGs and stained with mAb 7–239 α-Siglec-1-PE or matched isotype-PE control (AbD Serotec) at 4°C for 30 min. The mean number of Siglec-1 mAb binding sites per cell was obtained with a Quantibrite kit (Becton Dickinson) as previously described [9]. Samples were analyzed with FACSCalibur using CellQuest software to evaluate collected data.

Induction of Siglec-1 expression by plasmas of HIV-1-negative individuals and HIV-1-infected individuals before or after successful antiretroviral treatment was assessed on 2x10^5^ DCs derived from HIV-1-negative donors cultured for 24 h in the presence of 2% of each respective plasma. To block Siglec-1 induction by these plasmas, carrier-free recombinant B18R protein (eBioscience) was added at 2 μg/ml. DCs were labeled with mAb 7–239 α-Siglec-1-PE and quantified by Quantibrite. Basal values of Siglec-1 in DCs non-exposed to plasma were subtracted for each sample.

### Plasmids, viral stocks and cell lines

HEK-293 T and TZM-bl (obtained through the US National Institutes of Health [NIH] AIDS Research and Reference Reagent Program) were maintained in D-MEM containing 10% FBS, 100 U/ml of penicillin and 100 μg/ml of streptomicin.

HIV-1_NL4–3_ and VLP (VLP_HIV-Gag-eGFP_) stocks were generated by transfecting the molecular clones pNL4-3 and pGag-eGFP obtained from the NIH AIDS Research and Reference Reagent Program. HIV_NL4–3-Cherry_ was obtained by cotransfection of pCHIV and pCHIVmCherry [37] (kindly provided by Dr. B. Muller). HEK-293 T cells were transfected with calcium phosphate (CalPhos, Clontech) in T75 flasks using 30 μg of plasmid DNA. Supernatants containing virus or VLPs were filtered (Millex HV, 0.45 μm; Millipore) and frozen at −80°C until use. The p24^Gag^ content of the infectious viral stocks and VLPs was determined by an ELISA (Perkin-Elmer). HIV-1_NL4–3_ used in infectious assays was titrated employing the TZM-bl reporter cell line that expresses luciferase under control of the HIV-1 promoter, as described in [38].

### VLP and HIV-1 binding and uptake assays

Cells were pre-incubated at 4°C for 30 min with 10 μg/ml of the functional grade mAbα-Siglec-1 7–239, IgG1 isotype control (all from AbD Serotec) or left untreated. Binding was performed at 4°C while uptake was done at 37°C. To assess HIV-1_NL4–3_ binding and uptake, 4x10^5^ cells were pulsed with 970 ng of p24 for 4 h. After extensive washing, cells were lysed with 0.5% Triton X-100 to measure p24^Gag^ antigen content by an ELISA. To analyze viral degradation, cells were pre-incubated with 250 nM of bafilomycin A1 (Sigma) during 30 min at 37°C and then exposed to HIV_NL43_ in the presence of the drug or left untreated. To determine VLP binding and uptake, 2x10^5^ myeloid cells were pulsed with 10 ng of VLPs for 3 h and analyzed by FACS.

### HIV-1 *trans*-infection assays

Myeloid cells were treated and pulsed with HIV-1_NL4–3_ as described above. After extensive washing, cells were co-cultured with the reporter cell line TZM-bl at a ratio 1:1 or 5:1. Cells were assayed for luciferase activity 48 h later (BrightGlo luciferase system; Promega) in a Fluoroskan Ascent FL luminometer (Thermo Labsystems). Background values consisting of non-HIV-1 pulsed co-cultures were measured for each experiment. Of note, we chose the X4-tropic virus NL4-3 and short period co-culture assays to avoid productive *cis*-infection of myeloid cells and focus on *trans*-infection.

### Confocal microscopy analysis

4x10^5^ myeloid cells were pulsed with HIV-1_Gag-Cherry_ for 4 h at 37°C as previously described [9]. After extensive washing, cells were fixed and permeabilized (Fix & Perm; Invitrogen) and stained with mAbs α-Siglec-1 7–239 Alexa 488 (AbD Serotec), α-HLA-DR-Alexa 647 (Clone L243, Biolegend) and DAPI for 30 min. Cells were cytospun into coverslips and analyzed with an Ultraview ERS Spinning Disk System (Perkin-Elmer) mounted on a Zeiss Axiovert 200 M inverted microscope. Volocity software (Perkin-Elmer) was used to analyze microscopy images as in [13].

### Paraffinized tissues and immunoenzyme staining

Paraffinized tonsils from HIV-1 non-infected individuals were provided by the tissue bank of the National Center for Tumor Diseases (Heidelberg, Germany) and approved by the ethics committee of Heidelberg University (approval No. 206/2005). Immunoenzyme staining of Siglec-1 were performed on 2-μm paraffin sections of formalin-fixed tissues in principle as reported [39]. Antigen retrieval was achieved by steam cooking the slides in 10 mM citrate buffer (pH 6.1, Dako) for 30 min. 10% Earle’s balanced salt solution (EBSS, Sigma Aldrich) supplemented with 1% HEPES, 0.2% bovine serum albumin, and 0.1% saponin (all from Sigma) at pH 7.4 was used as washing and permeabilization buffer. Primary mAb dilutions with α-Siglec-1 7D2 (Novus Biologicals) were also prepared in this buffer and incubated overnight at 4°C. Slides were blocked in 15% sheep serum for 20 min and revealed by biotinylated sheep anti-mouse Ab for 30 min at RT. Immunoenzyme staining was performed with standard avidin-biotin anti-alkaline phosphatase techniques (Vectastain). Naphthol AS-biphosphate (Sigma) with New Fuchsin (Merck) was used as the substrate for alkaline phosphatase. Slides were viewed with an Olympus BX45 microscope. Tonsils were classified by an experienced pathologist as inflamed based on strong tissue infiltration of neutrophil granulocytes.

Paraffinized axillary and abdominal lymph nodes from an HIV/HCV co-infected patient were analyzed at the Pathology Department of HUGTIP. For immunohistochemistry, 4-μm paraffin-embedded sections were cut, deparaffinized and rehydrated through xylene and graded alcohols to water. Antigen retrieval was done immersing the slides for 40 minutes in EDTA Buffer in a water bath at 98°C. The staining was performed using as primary mAbs α-Siglec-1 7D2, α-CD4 (Clone SP35, Ventana Medical Systems) and α-CD20cy (Clone L26, DAKO) and the Ventana Discovery XT automated stainer (Ventana Medical Systems,) with ultraView Universal DAB Detection Kit. Of note, patient had been treated with two nucleoside reverse transcriptase inhibitors for 7 years and had stopped treatment for 4 years, when the first biopsy was performed. At that time point, HIV-1 plasma viral load was < 50 HIV-1 RNA copies/ml and CD4^+^ T cell-count was 485. Patient started antiretroviral treatment again with a protease inhibitor-based regimen and had a second biopsy one year later, when HIV-1 plasma viral load was <25 HIV-1 RNA copies/ml and CD4^+^ T cell-count was 511. At the second biopsy, HCV viral load was 641.144 UI/ml.

### Transcriptome RNA-seq analysis

RNA extraction from IFNα-treated DCs, macrophages and monocytes cells (1–6 x10^6^) was performed using RNeasy Mini kit (Qiagen). RNA extraction from sorted IFNα-treated Siglec-1 tonsillar cells was performed using RNeasy Micro kit (Qiagen). mRNA-Seq library preparation was done with TruSeq RNA sample prep kit, Illumina (starting with capture of polyA-containing transcripts), followed by cluster generation (TruSeq single-end cluster generation kit, Illumina) and high-throughput sequencing on Illumina HiSeq2000 at the Genomics Technology Facility, University of Lausanne. The 100 bp single-end reads obtained were cleaned before alignment as described in [40]. Cleaned reads were aligned to the human reference genome with STAR aligner [41] using the ensembl gene GRCh37 release 70 annotation file. The number of reads per gene was quantified with HTSeq-count v.0.6.1 [42] with parameters mode = union and type = exon. We obtained an average library size of 45072173 uniquely mapped reads. All downstream analyses were performed taking as gene expression values the log_10_ of the number of library size-normalized reads per kilobase of exonic sequence. A pseudo-count of 1 was added previous to the log_10_ transformation to avoid NA’s (impossible log transformation) and obtain a numerical value.

### Statistical analysis

We analyzed mean changes using a paired t-test, which was considered significant at *P* < 0.05. Mean changes of unpaired observations were assessed using the Man Whitney t-test, which was considered significant at *P* < 0.05. Significant mean changes from 100% of the data normalized to percentages were assessed with a one sample t-test, considered significant at *P* < 0.04. Pearson correlation tests were used to determine the level of association between Siglec-1 Ab binding sites per monocyte and VLP capture, HIV-1 capture, HIV-1 *trans*-infection, plasma viral load or CD4^+^ T-cell counts from HIV-1-infected individuals. All analyses and figures were generated with the GraphPad Prism v5.0b Software.

## Additional file

Additional file 1: Figure S1. Siglec-1 mediates VLP capture by IFNα-treated myeloid cells. A**.** Comparative binding of fluorescent VLPs to different myeloid cells previously exposed to IFNα. Cells were pulsed with VLPs for 3 h at 4°C and then assessed by FACS to measure the geometric mean fluorescence intensity (MFI). B**.** Relative binding of VLPs by different IFNα- treated myeloid cells pre-incubated with 10 μg/ml of the indicated mAbs. Values are normalized to the level of VLP bound by mock-treated cells (set at 100%). C. Uptake of fluorescent VLPs by myeloid cells exposed to IFNα. Cells were pulsed with VLPs for 3 h at 37°C and assessed by FACS to measure the geometric MFI. D**.** Relative uptake of VLPs by IFNα-treated myeloid cells pre-incubated with 10 μg/ml of the indicated mAbs. Values are normalized to the level of VLP captured by mock-treated cells. All panels show mean values and SEM from 2 experiments including cells from 6 donors. Statistical differences were assessed with a paired t test in A, and with a one sample t-test in B and D. **Figure S2.** VLP uptake is not augmented in either tonsillar BDCA1-negative cells or B cells exposed to IFNα. A. Uptake of VLPs by CD19–/CD3–/BDCA1–-tonsillar cells previously exposed to IFNα or left untreated. Cells were pre-incubated with 10 μg/ml of the indicated mAbs. Mean values and SEM from 2 experiments include cells from 5 donors. B. Uptake of VLPs by CD19^+^ tonsillar cells previously exposed to IFNα or left untreated. Cells were pre-incubated with 10 μg/ml of the indicated mAbs. Mean values and SEM include cells from 2 donors.
