# Supplementary material for: HIV-1 immune activation induces Siglec-1 expression and enhances viral trans-infection in blood and tissue myeloid cells
Source: Retrovirology. 2015 May 7;12:37. doi: 10.1186/s12977-015-0160-x (PMC4423124; doi:10.1186/s12977-015-0160-x)
Supplement: Additional file 1: Figure S1. — Siglec-1 mediates VLP capture by IFNα-treated myeloid cells. A. Comparative binding of fluorescent VLPs to different myeloid cells previously exposed to IFNα. Cells were pulsed with VLPs for 3 h at 4°C and then assessed by FACS to measure the geometric mean fluorescence intensity (MFI). B. Relative binding of VLPs by different IFNα- treated myeloid cells pre-incubated with 10 μg/ml of the indicated mAbs. Values are normalized to the level of VLP bound by mock-treated cells (set at 100%). C. Uptake of fluorescent VLPs by myeloid cells exposed to IFNα. Cells were pulsed with VLPs for 3 h at 37°C and assessed by FACS to measure the geometric MFI. D. Relative uptake of VLPs by IFNα-treated myeloid cells pre-incubated with 10 μg/ml of the indicated mAbs. Values are normalized to the level of VLP captured by mock-treated cells. All panels show mean values and SEM from 2 experiments including cells from 6 donors. Statistical differences were assessed with a paired t test in A, and with a one sample t-test in B and D. Figure S2. VLP uptake is not augmented in either tonsillar BDCA1-negative cells or B cells exposed to IFNα. A. Uptake of VLPs by CD19–/CD3–/BDCA1–-tonsillar cells previously exposed to IFNα or left untreated. Cells were pre-incubated with 10 μg/ml of the indicated mAbs. Mean values and SEM from 2 experiments include cells from 5 donors. B. Uptake of VLPs by CD19+ tonsillar cells previously exposed to IFNα or left untreated. Cells were pre-incubated with 10 μg/ml of the indicated mAbs. Mean values and SEM include cells from 2 donors. [file 12977_2015_160_MOESM1_ESM.pdf]

SUPPLEMENTARY FIGURE LEGENDS

Supplementary Figure 1

A. Comparative binding of fluorescent VLPs to different myeloid cells previously exposed to 1000 U/ml of IFNα for 48 h. Cells were pulsed with VLPs for 3 h at 4ºC and then assessed by FACS to measure the geometric mean fluorescence intensity (MFI). Data show mean values and SEM from two experiments including cells from six donors.

B. Relative binding of VLPs by different IFNα-treated myeloid cells that had been pre-incubated with 10 µg/ml of the indicated mAbs before VLP exposure for 3 h at 4ºC. Values are normalized to the level of VLP captured by mock-treated cells (set at 100%). Data show mean values and SEM from two experiments including cells from six donors.

C. Uptake of fluorescent VLPs by different myeloid cells previously exposed to 1000 U/ml of IFNα for 48 h. Cells were pulsed with VLPs for 3 h at 37ºC and assessed by FACS to measure the geometric mean fluorescence intensity (MFI). Data show mean values and SEM from two experiments including cells from six donors.

D. Relative uptake of VLPs by different IFNα- treated myeloid cells that had been pre-incubated with 10 µg/ml of the indicated mAbs before VLP exposure for 3 h at 37ºC. Values are normalized to the level of VLP captured by mock-treated cells (set at 100%), and *P* values are calculated with a one sample t-test. Data show mean values and SEM from two experiments including cells from six donors.

Supplementary Figure 2

A. Uptake of VLPs by $CD19^{-}/CD3^{-}/BDCA1^{-}$-tonsillar cells previously exposed to 1000 U/ml of IFNα or left untreated for 48 h. Cells were pre-incubated with 10 μg/ml of the indicated mAbs before VLP exposure for 3 h at 37ºC. Data show mean values and SEM from two experiments including cells from five donors.

B. Uptake of VLPs by $CD19^{+}$tonsillar cells previously exposed to 1000 U/ml of IFNα or left untreated for 48 h. Cells were pre-incubated with 10 μg/ml of the indicated mAbs before VLP exposure for 3 h at 37ºC. Data show mean values and SEM including cells from two donors.

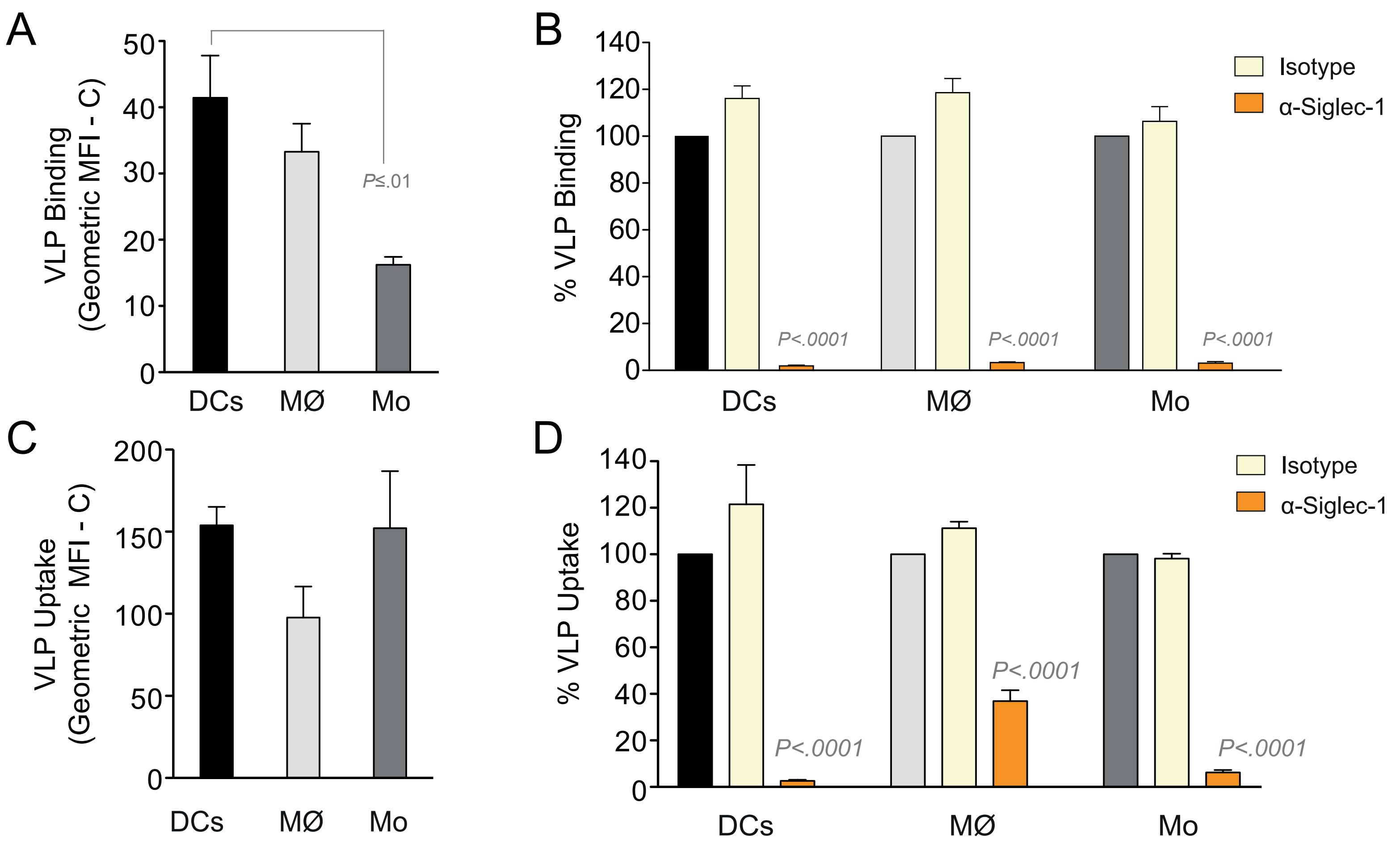

Supplemental Figure 1

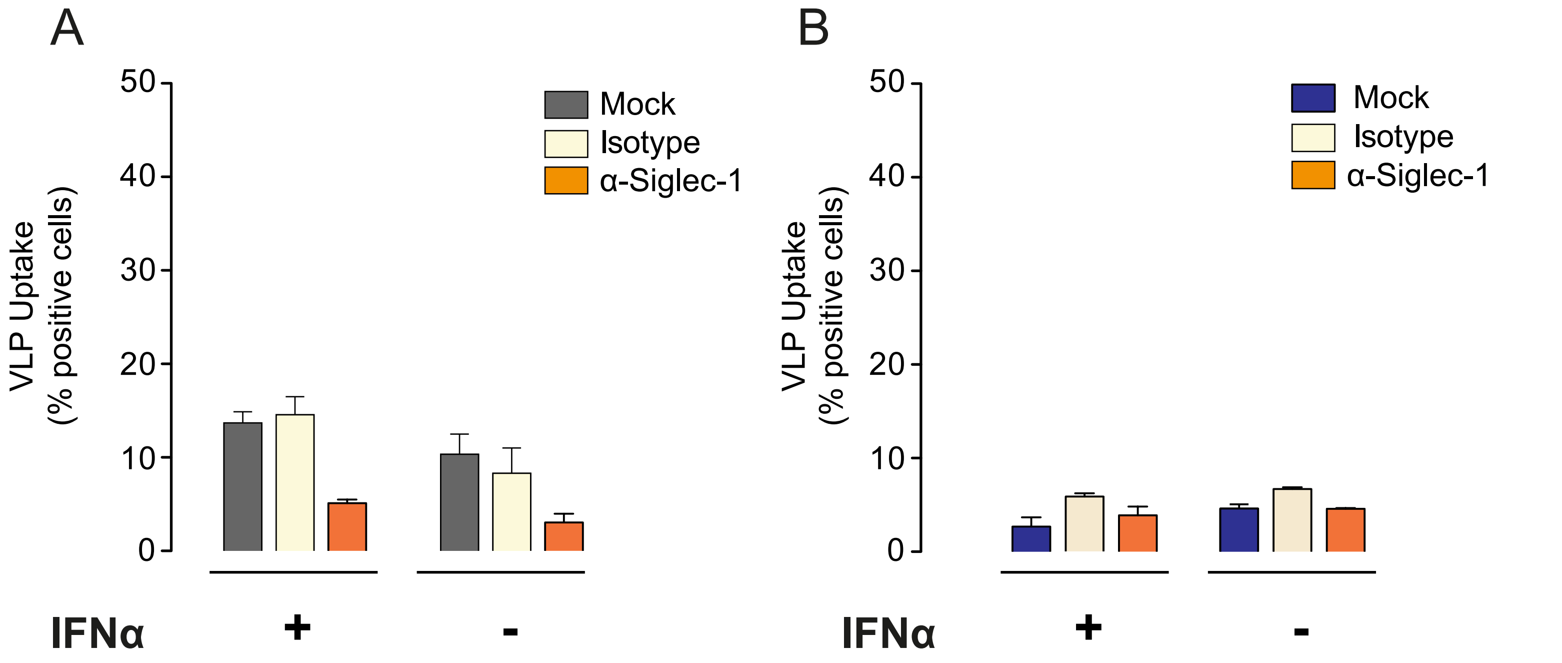

Supplemental Figure 2
